# A comparison of conductive ink usage optimization techniques used in fabrication of epidermal UHF radio frequency identification tags for medical and sensing applications

**DOI:** 10.1049/htl2.12051

**Published:** 2023-09-20

**Authors:** Dumtoochukwu Obiora Oyeka, John Batchelor, Rachel Saunders

**Affiliations:** ^1^ Department of Electronic Engineering at the University of Nigeria Nsukka Enugu Nigeria; ^2^ School of Engineering and Digital Arts University of Kent Canterbury Kent UK; ^3^ Department of Materials University of Manchester Manchester UK

**Keywords:** assisted living, body area networks, body sensor networks, health care, ink jet printing, radiofrequency identification, sensors

## Abstract

The aim of this work is to assess the performance of various inkjet printing techniques. These techniques are aimed at optimizing the volume of conductive ink used in the fabrication of inkjet printed Radio Frequency Identification tags. It is also possible that they can be used in fabricating other electronic and electromagnetic devices and structures. Three ink optimization approaches were examined viz. gridded (meshed) designs, conductive area trimming and selective ink deposition. The volume of conductive ink utilized in tag fabrication and the measured on‐body (forearm) read range of the tag were used to develop a figure of merit which determined the best printing approach. Although the longest read range was obtained from the tag with 48% conductive area trimming (Trim 1), the best figure of merit, that is, the tag with the best balance between measured read range and utilized conductive ink, was obtained from the tag that had its surface area trimmed by 65% (Trim 2). It is however suggested that optimum use of conductive ink would be achieved with a combination of 65% surface area trimming and selective ink deposition technique.

## Graphical abstract

This work investigates the effect of various ink conservation and optimisation techniques that can be employed in the fabrication of epidermal radio frequency identification tags. These techniques are compared by using a figure of merit and the optimal technique is identified.

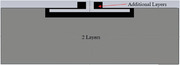

## 1 INTRODUCTION

Inkjet printing is becoming more attractive for the fabrication of electronic and electromagnetic devices including Radio Frequency Identification (RFID) tags [1] and [2], sensors [3, 4], smart devices [5], for healthcare applications [6, 7], as well as antennas for the use in fifth‐generation technologies [8, 9]. This traction being gained by inkjet printing is due to some of its inherent benefits which include ease of fabrication, flexibility, and ability to conform with surfaces (substrates) [10]. For body‐mounted devices, in order to ensure the best user experience, flexibility is particularly desirable because this translates to less intrusion and more comfort for the user. In fact, the user should be unaffected by the presence of such devices.

A challenge to reaping the benefits of inkjet printing is the increasing cost as the volume of the utilized conductive ink increases [11, 12]. This challenge has led to some creative means of reducing the volume of ink (hence cost reduction) used in inkjet printing while maintaining good tag read range. Some popular techniques used to reduce ink usage per tag considered in this work include trimming off of low current density areas of the tag [13], selective deposition of ink in high current concentration areas of the RFID tag and use of meshed designs [14]. These are all geared towards ensuring favourable competition between inkjet printing and other conventional fabrication techniques.

This work aims to assess the performance (obtained read range) of these techniques while also considering the volume of ink each utilizes. These two factors were then used to develop a figure of merit to establish the best ink usage optimization approach.

## 2 METHODS

**FIGURE 1:**
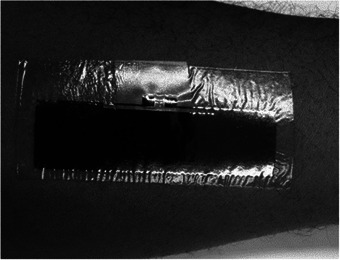
Inkjet printed epidermal tag placed on the arm.

The tag, Figure 1, presented in [15] was used for this study. It resonates at the european union (EU) ultra high frequency (UHF) RFID Frequency band of 865 to 868 MHz. It also has wide bandwidth for operation in other bands. More details about the tag including fabrication are presented in [16]. The conductive ink used was Sun Chemicals’ ‘Silver Nanoparticle ink’ while Simulia CST Studio Suite [17] was used for simulation. Measurements were carried out using with the Tagformance kit [18].

**FIGURE 2:**
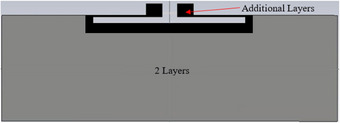
Additional ink deposition regions.

The selective deposition of ink during tag fabrication was guided by simulation results. This was because of the possibility of visualizing high current density areas which were around the tag slot and feedline. These areas were consequently considered critical to the tag's operation, hence the use of more ink volume there to ensure adequate conductivity as well as attainment of skin depth requirements at the tag's operating frequency [19]. This is shown in Figure 2.

This ink optimization approach had three types: two full layers with one extra layer around the slot, ports, and feedline (2F + 1), two full layers with two extra layers around the slot, ports, and feedline (2F + 2), and two full layers with three extra layers around the slot, ports, and feedline (2F + 3).

**FIGURE 3:**
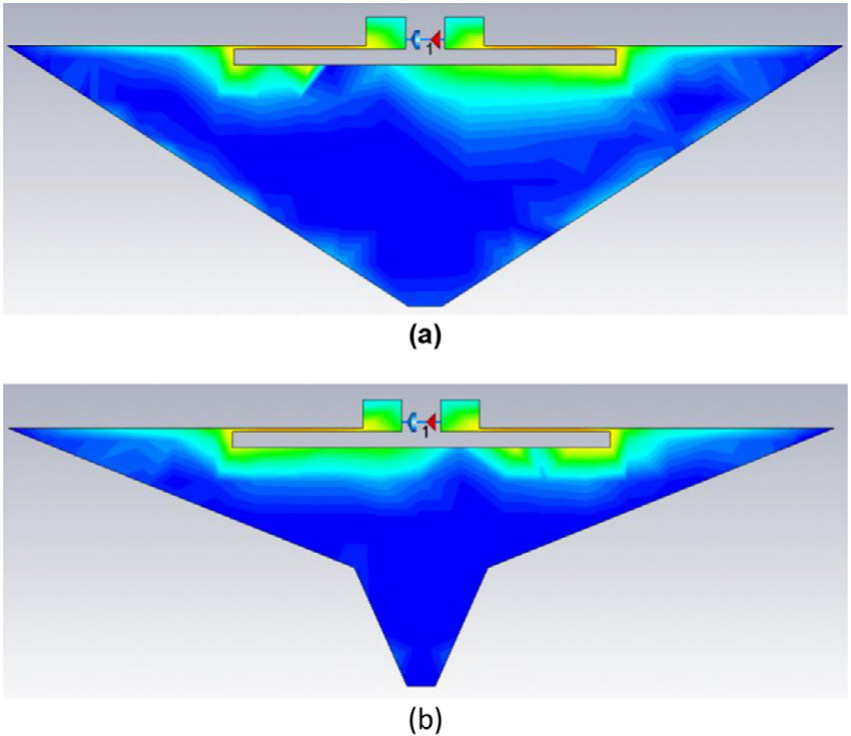
Trimmed tags: (a) Trim 1% to 48% trimmed. (b) Trim 2% to 65% trimmed.

Provided that the high current density areas of the tag are not severely tampered with hence restricting the flow of current, the overall surface area of the tag can be reduced without affecting tag functionality. This means the low current density areas can be trimmed off leaving mostly the high current density areas to be printed. More conductive area can be trimmed off to save more ink even though this comes with a tradeoff of reduced read range. Two samples of this technique were examined: the first one had its surface area decreased by 48% (‘Trim 1′) while the second one had its surface area reduced by 65% (‘Trim 2′). These are illustrated in Figure 3.

**FIGURE 4:**
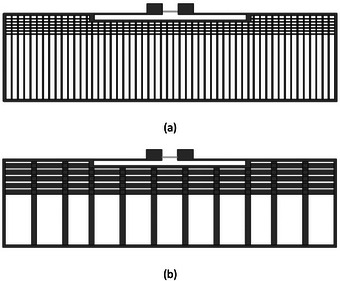
Gridded designs. (a) Grid 1. (b) Grid 2.

Finally, gridded designs of the kind discussed in [11] were considered. Gridded designs also known as meshed designs have been previously used by different researchers for the purpose of reduction of the area occupied by an antenna, conformance with immediate environment as well as reduction in fabrication material volume. For this study, two samples of gridded antenna designs were used: ‘Grid 1′ which is a mesh with 0.2‐mm wide vertical tracks with 5‐mm spacing with four horizontal tracks in the high surface current area located around the feedline and slot area and ‘Grid 2′ comprising of 1‐mm‐wide vertical tracks with 5‐mm spacing and four horizontal tracks in the area around the slot and feedline. The gridded tag designs are shown in Figure 4.

All tags were printed with a drop spacing of 20 μm; hence, a direct comparison can be made between each of them.

All read range measurements were taken with the tags placed on the forearm.

## 3 RESULTS AND DISCUSSION

**FIGURE 5:**
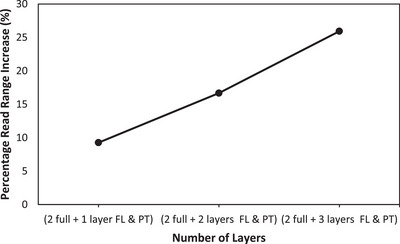
Percentage read range of selectively deposited conductive ink tags (FL and PT = feedline and ports/slot).

A plot of the change in read range (when placed on the forearm) by percentage between the various selectively deposited ink tags and a reference three full conductive ink layers tag is shown in Figure 5.

From Figure 5, there is a clear linear relationship between read range and volume of deposited ink. This is expected as increasing the volume of ink especially in the high current density areas improves conductivity, hence current flow, skin depth performance, as well as tag efficiency. A single extra layer around the slot, ports, and feedline resulted in an increase by 9% in the original read range of the tag and this trend was gradual till a 26% increase was obtained for the tag with three extra layers on the slot, ports, and feedline region (2 full + 3 layers).

**FIGURE 6:**
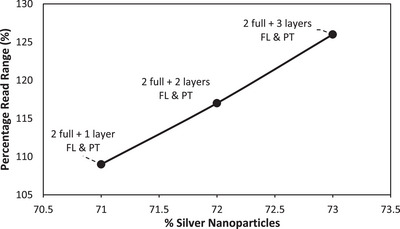
Correlation between utilized ink and achieved read range of selectively deposited conductive ink tags with reference to a full three‐layer tag.

Further study established a relationship between the obtained read range and the volume of silver nanoparticles used for the fabrication of the tags. This was achieved by using the ink manufacturer's provided silver particle concentration of the ink (35%) and the dimensions of the printed tags. The total mass of conductive ink used to make the full transfer tattoo tag was determined to be 0.217 g of which the silver nanoparticles consist of 0.076 g. The resulting values also agree with the trend reported above with each subsequent added layer leading to a 1% increase in the volume of silver nanoparticles used hence overall conductive ink volume. These results are presented in Figure 6.

**FIGURE 7:**
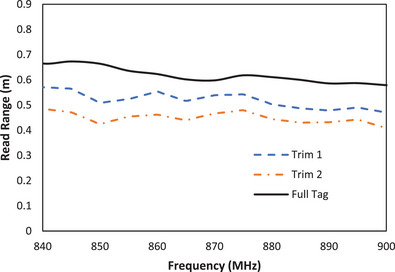
Measured read range of tags with conductive area trimmed.

Figure 7 shows the effect of trimming the conductive area of the tag as a means of optimizing ink usage on the read range of the tag.

The reduction of the surface area of the tag led to a reduction in the tag efficiency which in turn resulted in a reduction in tag read range. This reduction in efficiency was more pronounced as the trimmed off part of the tag was increased. ‘Trim 1′ with 48% of its surface removed showed better read range (53 cm) than ‘Trim 2′ (45 cm read range) with 65% of its conductive area trimmed off. It is noteworthy that as the removed conductive parts of the tag increased, there was also reduction in the measured backscattered power and an increase in the transmitted power requirement for tag activation (to attain its threshold power). This is also true for the other fabrication techniques presented in this work.

**FIGURE 8:**
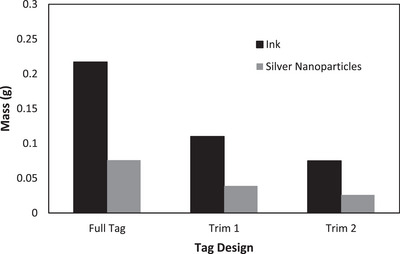
Mass of silver nanoparticles and ink utilized per trimmed tag.

The total mass of silver nanoparticles used for the fabrication of ‘Trim 1′ tag was calculated to be 0.039 g—a 49% decrease in total silver nanoparticles used to fabricate the full three‐layer tag. For ‘Trim 2′, 0.026 g of silver nanoparticles was used. This represented 34% of the quantity of silver nanoparticles used to fabricate the original tag. Figure 8 shows a graphical representation of this.

**FIGURE 9:**
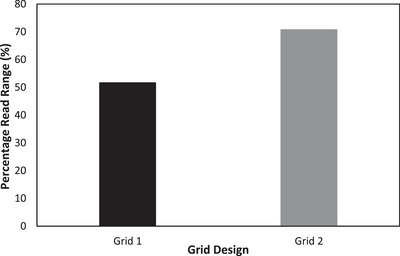
Percentage read range of gridded tags.

The percentage change in the mesh tag's read range is illustrated in Figure 9. It was observed that ‘Grid 2′ tag with the wider grid widths had a measured read range of about 71% of the full tag, while ‘Grid 1′ had about 48% decrease in the original measured read range.

Significantly less ink was utilized in fabricating the gridded tags than was for the full tag. ‘Grid 1′ utilized only 0.021 g of silver nano particles (28% of the mass of silver nanoparticles utilized for the tag with three full layers), while 0.03 g (44% of the full tag) of silver nanoparticles was used to fabricate ‘Grid 2′—a significant decrease in volume of ink used albeit with a read range trade‐off.

Table 1 summarizes the measured read range and calculates utilized silver nanoparticles by each presented tag with reference to the full tag. The figure of merit is the ratio of the achieved percentage read range to the percentage conductive ink used for the fabrication of the particular tag with reference to the full three‐layer tag. With this method, a high figure of merit is desired.

**TABLE 1:** Comparison of ink usage minimization techniques.

Tag design	Ink mass (%)	Ag nanoparticle mass (%)	Read range (%)	Fig of merit
Trim 1	51	51	88	1.72
Trim 2	35	34	75	2.14
Grid 1	27	28	52	1.92
Grid 2	44	44	71	1.61
2F + 1	71	71	109	1.54
2F + 2	72	72	117	1.63
2F + 3	73	73	126	1.73

The results from Table 1 indicate that the best read ranges were obtained from tags which had two full layers with extra layers on the slot, ports, and feedline region. However, the drawback to this is that it comes at a cost of higher utilized conductive ink volume. As a result of this, these tags have lower figure of merit compared to the other tags with the highest being 1.73 obtained for the tag with extra three layers of conductive ink on the slot, ports, and feedline area (2F + 3). The best measured read range was from the ‘Trim 1′ tag with 88% of the read range of the original (3‐layer tag) measured. The figure of merit of 2.14, which is the best, was obtained from ‘Trim 2′ as this tag provided the best balance between obtained read range and ink volume use. Even though ‘Grid 1′ used the least volume of ink (27%), it also resulted in the worst read range of all the tags in the study (52%). The measured read range for ‘Grid 2′ was 71% of the read range of the original tag with 44% of the conductive ink utilized for the full three‐layer tag. The robustness of these gridded designs could also be of concern because due to the thin nature of the grids, there might be a degradation in performance if they break especially in high current density areas.

With the results in Table 1, it can be concluded that the best trade‐off between obtained read range and ink volume utilization is a combination of ‘Trim 2′ and ‘2F + 1′.

**FIGURE 10:**
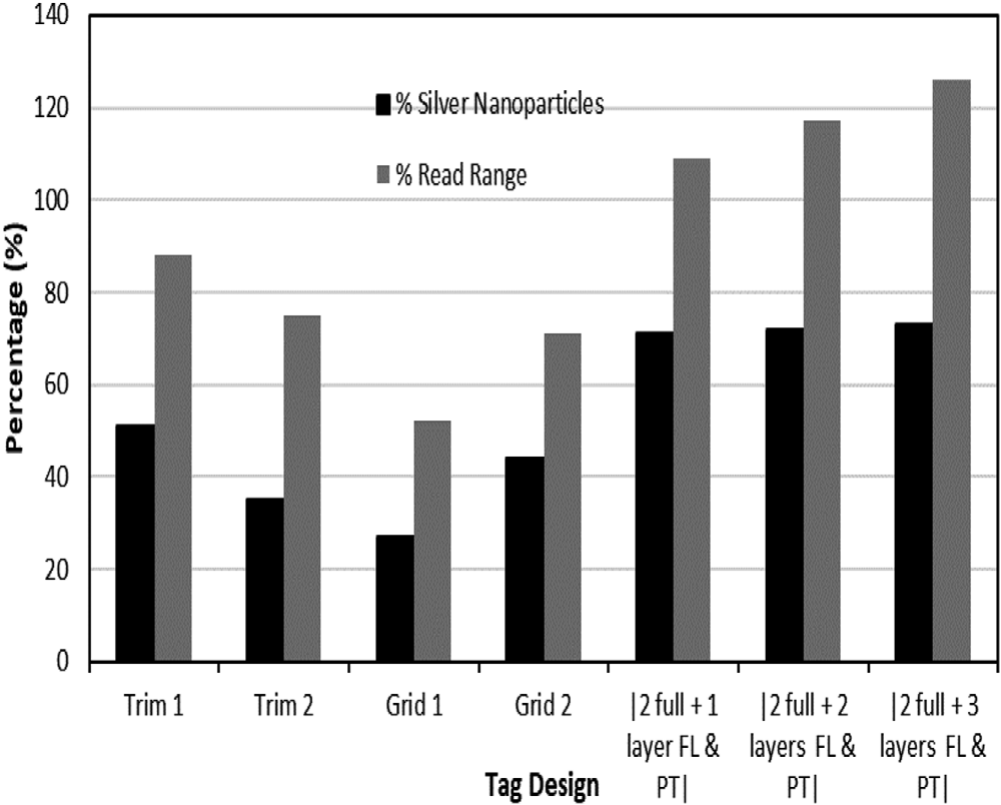
Read range and utilized ink volume comparison.

A direct comparison between obtained read range and utilized ink volume for all the presented samples is presented in Figure 10.

## 4 CONCLUSION

Three ink usage optimization techniques have been examined in this work and the results obtained indicate that reasonable read range can be obtained from the tags even with reduced volume of conductive ink. The greatest read range was obtained when the surface area of the tag was trimmed by 48% while the least ink was used in fabricating the gridded design, ‘Grid 1′, even though tags of this kind could have robustness concerns. Some tags however had a better balance between the obtained read range and ink volume used. This was evidenced by their high figure of merit. Best among these was the ‘Trim 2′ tag with a figure of merit of 2.14. A good approach could be to employ a printing style that combines techniques identified in this work that achieved good read range and those that used minimal ink. An example of this is a combination of cutting off some parts of the tag as shown in ‘Trim 2′ and printing two full layers of conductive ink with an extra layer on the feedline and slot area.

## CREDIT CONTRIBUTION STATEMENT

Dumtoochukwu Oyeka: Conceptualization; Data curation; Formal analysis; Investigation; Methodology; Writing – original draft; Writing – review & editing. John Batchelor: Methodology; Project administration; Resources; Supervision; Writing – review & editing. Rachel Saunders: Investigation; Methodology; Resources; Writing – review & editing.

## CONFLICT OF INTEREST STATEMENT

The authors declare no conflict of interest.

## Data Availability

Data sharing not applicable to this article as no datasets were generated or analyzed during the current study.
